# Factors Associated With Early Recurrence in Non‐Surgically Managed Primary Spontaneous Pneumothorax

**DOI:** 10.1002/wjs.70008

**Published:** 2025-07-21

**Authors:** Sercan Aydin, Seda Kahraman Aydin, Dilara Gursoy, Ayse Gul Ergonul, Tevfik Ilker Akcam, Kutsal Turhan, Ufuk Cagirici, Alpaslan Cakan, Omer Faruk Dadas

**Affiliations:** ^1^ Department of Thoracic Surgery Izmir Democracy University Buca Seyfi Demirsoy Education and Research Hospital Izmir Turkey; ^2^ Department of Thoracic Surgery Ege University Faculty of Medicine Izmir Turkey; ^3^ Department of Thoracic Surgery Acibadem Kent Hospital Izmir Turkey; ^4^ Department of Biostatistics and Medical Informatics Ege University Faculty of Medicine Izmir Turkey

**Keywords:** bleb, bullae, disease‐free survival, primary spontaneous pneumothorax, smoking, surgical treatment

## Abstract

**Background:**

The indications for surgery in primary spontaneous pneumothorax (PSP) at first episode are clearly defined. Despite the indications, some patients avoid surgical treatment. This study examines the factors affecting early recurrence in patients who have not undergone surgical intervention.

**Methods:**

This retrospective and multicenter study was conducted between January 2018 and June 2023. The impact of demographic characteristics, inflammatory response, smoking habits before and after the episode, treatment modality, duration of chest tube drainage, and quantitative measurements of bullae–bleb structures on the risk and timing of recurrence was examined in 117 cases of first episode PSP.

**Results:**

Age, sex, inflammatory response indicators, pre‐episode smoking history, pneumothorax volume, and bullae size were not associated with earlier recurrence. The detection of bullae–blebs on computed tomography in first episode PSP and smoking post‐episode in non‐surgically treated cases were correlated with earlier recurrence (*p* = 0.031; *p* = 0.019, respectively). In cases that surgical intervention had not been performed, a prolonged duration of chest tube drainage correlated with increased recurrence‐free survival and decreased recurrence risk (*p* = 0.002).

**Conclusion:**

In cases of first episode PSP, surgical intervention may be advised when bullae–bleb formations are detected via tomography. Patients who did not undergo surgical treatment during the first episode and smoke after discharge are at higher risk of earlier recurrence. Prolonged chest tube drainage duration reduces the risk of earlier recurrence in first‐episode PSP cases that have not undergone surgical intervention. This finding suggests that extended drainage is not a disadvantageous outcome for cases hesitant to undergo surgical treatment.

## Graphical abstract

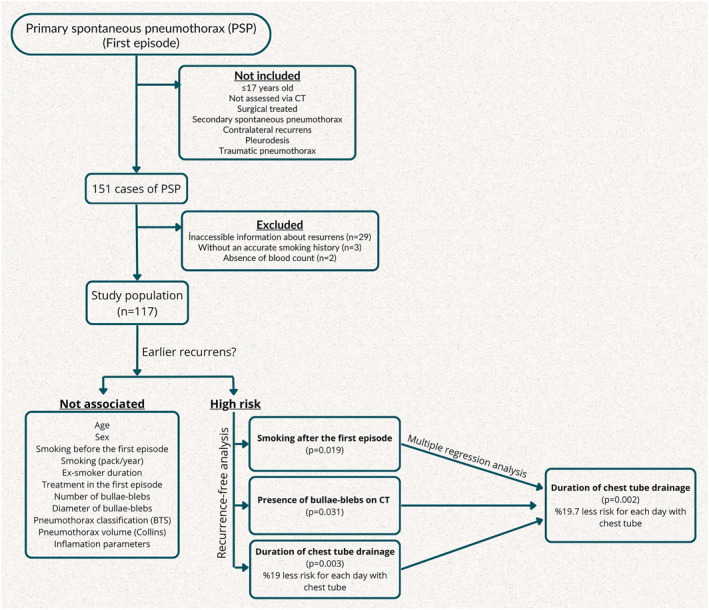

## 1 Introduction

Primary spontaneous pneumothorax (PSP) is characterized by the collapse of the lung and the presence of air in the pleural cavity without any trauma or significant underlying pulmonary pathology. It is more common among young, tall, and slender patients. Treatment modalities for PSP include observation, tube thoracostomy, and video‐assisted thoracoscopic (VATS) wedge resection. The mechanism of PSP is typically elucidated by the rupture of small air sacs known as bullae or blebs on the lung’s surface. Researchers predominantly agree that smoking, low body mass index, and genetic predisposition are the primary risk factors for PSP. Inflammatory processes induced by smoking damage lung tissue and promoted the development of pneumothorax. Studies have shown that the risk of recurrent pneumothorax is higher in individuals who were active smokers prior to the episode, compared to those who had quit smoking or had never smoked before the episode [1, 2].

Several studies have been conducted to investigate the relationship between pneumothorax and inflammatory processes. Studies indicate that pneumothorax enhances the inflammatory response in both the blood and pleura [3]. The appearance of pulmonary bronchiolitis and the increased inflammatory cell count in wedge resection specimens of PSP cases suggest that inflammation may contribute to the etiology of PSP [1]. Building on this evidence, the present study aims to investigate whether inflammatory markers can predict recurrence in patients experiencing a first episode of PSP that was managed non‐surgically. We specifically evaluated several inflammation‐related blood parameters—white blood cell (WBC) count, systemic immune‐inflammation index (SII), neutrophil‐to‐lymphocyte ratio (NLR), platelet‐to‐lymphocyte ratio (PLR), and systemic inflammatory response index (SIRI)—all of which are easily obtained through routine blood tests and have been increasingly studied in recent years for their diagnostic and prognostic value in various medical conditions [4, 5, 6]. Research on the recurrence of primary spontaneous pneumothorax (PSP) typically includes data from all treatment approaches, evaluates the risk of recurrence, and accounts for smoking history solely in the pre‐episode period. In clinical practice, a significant uncertainty for patients recommended for surgery during first episode (FE) is the timing of potential recurrence. This uncertainty may affect the decision of numerous patients for surgery following the FE. Our study examined the factors influencing the recurrence of spontaneous pneumothorax managed without surgical intervention, utilizing only observation or tube thoracostomy. Unlike many reports in the literature that include patients who underwent surgery, our research specifically focused on patients managed exclusively with non‐surgical approaches. Not only was recurrence risk investigated, but also, as a novel aspect, recurrence‐free survival (RFS) was investigated. Based on the hypothesis that inflammatory response may affect the quality of healing of bullae structures, we analyzed the effects of inflammatory response, smoking before and—as a novel aspect—after the episode, treatment method, duration of chest tube drainage (DCT), and quantitative measurements of bullae‐bleb structures on recurrence risk and RFS.

## 2 Methods

Our retrospective study was conducted following the approval from the local ethics committee, dated February 28, 2024, and assigned the number 2024/242. The data of PSP patients treated at two centers from January 2018 to June 2023 were analyzed.

### 2.1 The Criteria for Inclusion

The study included cases of first episode PSP in individuals over 17 years of age, diagnosed by thorax computerized tomography (CT), and subsequently observed in the thoracic surgery clinic.

### 2.2 The Criteria for Exclusion

**FIGURE 1:**
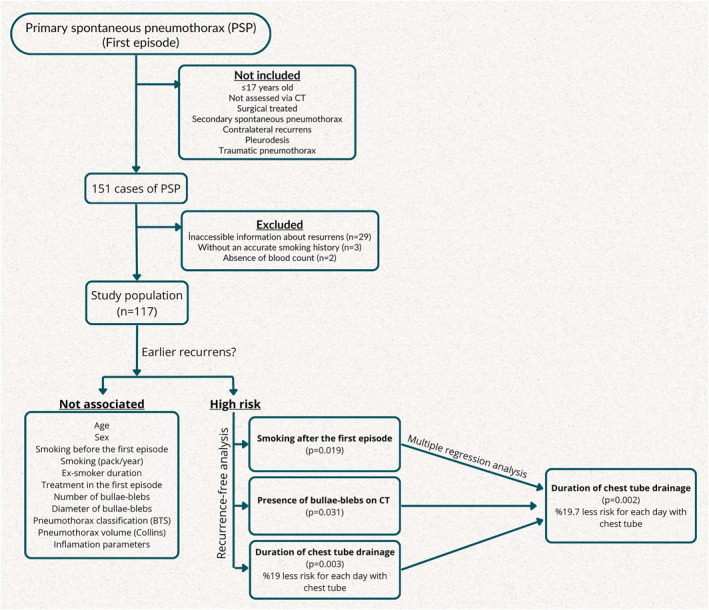
Flowchart with exclusion criteria and analysis results.

Cases not assessed via CT during the FE, treated with VATS wedge resection for any indication in the FE, classified as secondary spontaneous pneumothorax based on radiological data or medical history, identified with contralateral recurrence during follow‐up investigations, undergoing pleurodesis for any reason during the FE, and cases of traumatic pneumothorax were not included in the study. In the present study, the aim was to obtain an isolated patient group by excluding PSP cases who underwent wedge resection which is considered the definitive treatment for PSP. The objective was to evaluate the effects of smoking after the initial episode, the patient's inflammatory response, and other variables on the untreated lung, and consequently, their impact on the risk and duration of recurrence. The exclusion of contralateral pneumothorax cases aimed to enhance the understanding of inflammatory pathologies in bullae–bleb healing. Following the initial exclusions, from the remaining 151 patients 29 cases with inaccessible information during the recurrence investigation, 3 cases without an accurate smoking history, and 2 cases lacking hemogram tests were excluded from the study. Considering the possibility that patients might present to a different hospital in the event of a recurrence, recurrence status was checked through the national patient data platform. However, in accordance with personal data protection laws, some patients do not consent to share their medical information. Patients whose data could not be accessed are those for whom recurrence could not be confirmed through either our hospital's information system or the national database. The exclusion criteria were presented as a flowchart (Figure 1).

The analysis was conducted using data from 117 patients. Demographic characteristics, smoking history prior to FE, hemogram parameters, pneumothorax volumes (calculated using the Collins method) [7], pneumothorax sizes (classified according to the British Thoracic Society(BTS)) [8], presence of bullae–blebs on the initial CT, quantity, and total diameters of bullae, treatment methods administered during the FE, DCT, and smoking history following the FE were documented. The reference date for calculating the RFS was established as August 2023. The inclusion and exclusion criteria were meticulously defined to create a specific study population aimed at elucidating the local and general factors affecting recurrence in patients who underwent no surgical treatment.

In our study, thoracic drains were routinely removed on the second day following the cessation of air leakage. Clamp trials were not performed as part of the standard practice. Air drainage exceeding 7 days after tube thoracostomy for FE was defined as prolonged air drainage. In the study population, patients with chest tube durations shorter than 7 days were not recommended surgery for any reason, whereas those with durations longer than 7 days were recommended surgery but declined it, and therefore remained part of the study population as they were not operated on. Since it was not suitable for the intended analysis, the data of chest tube duration was evaluated numerically rather than categorically.

Reference values for hemogram parameters were determined as follows: 4–10 × 10^3^/mm^3^ for leukocytes, 1.51–7.07 × 10^3^/mm^3^ for neutrophils, 0.65–2.80 × 10^3^/mm^3^ for lymphocytes, 0.12–1.20 × 10^3^/mm^3^ for monocytes, and 150–450 × 10^3^/mm^3^ for platelets. The neutrophil‐to‐lymphocyte ratio, the derived neutrophil‐to‐lymphocyte ratio (dNLR), the lymphocyte‐to‐monocyte ratio (LMR), the platelet‐to‐lymphocyte ratio (PLR), the systemic inflammation index, the systemic inflammation response index, and the systemic inflammation aggregate index (AISI) were utilized as inflammation markers in the study. Blood samples obtained from patients upon their initial hospital admission during the first episode, prior to chest tube insertion, were assessed to determine the impact of the inflammatory response on recurrence.

### 2.3 Statistical Analysis

Descriptive statistics were used to analyze the data, including measures such as mean, standard deviation, median, minimum, maximum, frequency, and percentage values. For cases with recurrence, the follow‐up period is the time that has passed from the first pneumothorax episode until the recurrence. In cases without recurrence, it is defined as the period from the first pneumothorax until August 2023. Kaplan–Meier curves were used to estimate recurrence‐free survival probabilities for categorical variables. The Log‐rank test was employed to test for significant differences between categories of each categorical variable. The effect of numerical variables on recurrence‐free survival was evaluated using the Cox regression model. In the univariate tests, variables with a *p*‐value < 0.10 and those considered clinically significant were included in the multiple Cox regression model to evaluate if they were risk factors for spontaneous pneumothorax. Hazard ratios and 95% confidence intervals were calculated for each risk factor. Data were analyzed using IBM SPSS Statistics 25.0 (IBM SPSS Statistics for Windows, Version 25.0. Armonk, NY: IBM Corp.). A significance level of 0.05 was used for all analyses.

## 3 Results

In the study of 117 patients, 109 (93.2%) were male. The mean age of the population was 28.8 ± 8.9 (17–58) years. Fifty‐nine cases (50.4%) were managed for left pneumothorax. Seventy (59.8%) of the cases were active smokers at the time of the FE. In radiologic assessments, the majority of cases exhibited minimal pneumothorax volume based on BTS criteria, with a mean pneumothorax volume of 47 ± 40 (1–100) cm^3^ according to the Collins method. Sixty‐two patients (53%) exhibited a bullae–bleb structure on CT. In the FE, 96 (82.1%) of the patients received treatment via a chest tube. Thirty‐four of these patients (35.4%) were individuals who were offered surgery because of prolonged air drainage but declined the procedure. The average DCT was 6 ± 2.4 (2–15) days. Using data retrieved from the hospital information system and the national patient database after patient discharge, it was determined that 69 (59%) of the patients discharged following the first episode (FE) continued smoking afterward. Seventy‐three cases (62.4%) experienced recurrence, and VATS wedge resection was performed in 48 of these cases. The demographic characteristics of the cases are presented in Table 1. Our study investigated the correlation between inflammatory response parameters documented in literature and the recurrence of the pneumothorax, based on the hypothesis that the inflammatory response may influence RFS. The analyzed parameters and their mean values are presented in Table 2.

**TABLE 1:** Demographic characteristics.

Characteristics		Frequency (*n*)	(%)
Mean age		28.8 ± 8.9 (17–58)	
Sex	Male	109	93.2
	Female	8	6.8
Pneumothorax side (first episode)	Right	58	49.6
	Left	59	50.4
Smoking habits (before first episode)	Smoker	70	59.8
	Ex‐smoker	21	17.9
	Never smoked	26	22.2
Smoking history (pack‐years)		13.3 ± 11.7 (1–64)	
Ex‐smoker duration (year)		6 ± 6.3 (1–20)	
Pneumothorax classification	Small	71	60.7
	Large	46	39.3
Pneumothorax volume (cm^3^)		47 ± 40 (1–100)	
Bulla–bleb on CT	Absence	55	47
	Single	11	9.4
	Multiple	51	43.6
Bullae–bleb total diameters (mm)		45 ± 34 (3–150)	
Treatment in the first episode	Chest tube	96	82.1
	Observation	21	17.9
Duration of chest tube drainage (day)		6 ± 2.4 (2–15)	
Smoking after the first episode	Smoker	69	59
	Non‐smoker	48	41
Treatment during recurrence	No recurrence	44	37.6
	VATS	48	41
	Chest tube	19	16.2
	Observation	6	5.1
Recurrence free survival (month)		26 ± 7.2 (1–138)	
Total		117	100

**TABLE 2:** Mean and standard deviation of inflammatory parameters and blood count components.

Characteristics	Mean ± STD (min‐max)
WBC (×10^3^/mm^3^)	9.7 ± 3.5 (2.6–19.8)
WBC‐N (×10^3^/mm^3^)	3.1 ± 1.1 (0.8–7)
Neutrophil (×10^3^/mm^3^)	6.6 ± 3.2 (1.8–17.6)
Lymphocyte (×10^3^/mm^3^)	2.3 ± 0.9 (0.4–5.6)
Monocyte (×10^3^/mm^3^)	0.7 ± 0.3 (0.1–1.9)
Platelet (×10^3^/mm^3^)	241.7 ± 56.4 (45–433)
NLR	3.6 ± 3 (0.8–18.6)
dNLR	2.5 ± 2.1 (0.6–16.2)
LMR	3.6 ± 1.5 (0.6–8.1)
PLR	125 ± 66.6 (47.2–453.4)
SII	877.5 ± 817 (197.1–4322)
SIRI	2.5 ± 3.1 (0.4–29.7)
AISI	613.3 ± 725.4 (82.7–5433.1)

Abbreviations: AISI, Systemic inflammation aggregate index [(neutrophils × platelets × monocytes/lymphocytes)]; dNLR, Derived neutrophil to lymphocyte ratio (N/(WBC‐N)); LMR, Lymphocyte to monocyte ratio; NLR, Neutrophil to lymphocyte ratio; PLR, Platelet lymphocyte ratio; SII, Systemic immune inflammation index [(platelet count × neutrophil count)/lymphocyte count]; SIRI, Systemic inflammatory response index [(neutrophil count × monocyte count)/lymphocyte count].

### 3.1 Recurrence‐Free Survival Analysis for Categorical Variables

The patient's sex, smoking history before the FE, treatment method administered during the FE, quantity of bullae‐blebs on CT, and the size of pneumothorax based on BTS were not identified as significant factors affecting the RFS. Patients with bullous lesions visible on CT were at higher risk for early recurrence (*p* = 0.031). Recurrence was observed in 50 out of 69 patients (72.5%) who smoked post‐episode period, whereas it was observed in 23 out of 48 patients (47.9%) who did not smoke. In other words, 50 of the 73 PSP cases (68.5%) with recurrence were patients who smoked after the initial episode. According to the recurrence‐free survival analysis patients who smoked after the FE demonstrated a significantly elevated risk for early recurrence compared to non‐smokers (*p* = 0.019) More specifically, patients who smoked after the first episode demonstrated a markedly shorter median recurrence‐free survival time (14.0 months) compared to non‐smokers (48.0 months), highlighting the potential impact of post‐episode smoking behavior on early recurrence (Table 3).

**TABLE 3:** Results of recurrence‐free survival analysis for categorical variables.

Characteristics		Estimate	STD	Lower bound	Upper Bound	*p*
Sex	Male	26,000	7736	10.838	41.162	0.661
	Female	20,000	—	—	—	
Smoking before the first episode	Smoker	26,000	15,308	0.001	56.004	0.982
	Ex‐smoker	24,000	3024	18.074	29.926	
	Never‐smoked	32,000	16,629	0.001	64.593	
Smoking after the first episode	Smoker	14,000	7839	0.001	29.364	***0.019***
	Non‐smoker	48,000	—	—	—	
Treatment in the first episode	Chest tube	24,000	7006	1.268	37.732	0.110
	Observation	84,000	48,263	0.001	178.595	
Bullae‐blebs on CT (first episode)	Absence	47,000	17,015	13.651	80.349	***0.031***
	Exist	12,000	9427	0.001	30.476	
Number of bullae‐blebs	Single	26,000	17,655	0.001	60.603	0.699
	Multiple	12,000	6238	0.001	24.226	
Pneumothorax classification (BTS)	Small	24,000	9752	4.885	43.115	0.738
	Large	28,000	7474	13.351	42.649	

*Note:* Statistically significant results are indicated in bold and italics.

### 3.2 Recurrence‐Free Survival Analysis for Numerical Variables

Age, smoking history prior to FE, duration after smoking cessation, total diameter of bullae–bleb structures, pneumothorax volume, and any inflammation parameter were not determined to have an impact on RFS. The analysis indicated that duration of chest tube drainage significantly affected RFS. The recurrence risk decreased by 19% for each additional day of chest tube drainage (HR = 0.810; *p* = 0.003) (Table 4).

**TABLE 4:** Recurrence‐free survival analysis for numerical variables.

				95.0% CI for exp(B)	
Characteristics	B	Sig.	Exp(B)	Lower	Upper
Age	−0.008	0.557	0.992	0.965	1.019
Smoking (pack years)	−0.004	0.747	0.996	0.972	1.021
Ex‐smoker duration (years)	−0.045	0.336	0.956	0.872	1.048
Duration of chest tube drainage (day)	−0.211	***0.003***	0.810	0.706	0.929
Bulla–bleb total diameters (mm)	−0.002	0.618	0.998	0.990	1.006
Pneumothorax volume (cm^3^)	0.001	0.906	1.001	0.993	1.008
NLR	−0.016	0.672	0.984	0.913	1.061
dNLR	−0.003	0.955	0.997	0.896	1.109
LMR	0.044	0.565	1.045	0.900	1.213
PLR	0.000	0.890	1.000	0.997	1.004
SII	0.000	0.828	1.000	1.000	1.000
SIRI	−0.017	0.639	0.983	0.917	1.054
AISI	0.000	0.642	1.000	1.000	1.000

*Note:* Statistically significant results are indicated in bold and italics.

### 3.3 Multiple Regression Analysis on Recurrence‐Free Survival

A multiple regression model was established including the DCT and the presence of bulla–bleb structures on CT, both of which were statistically significant in our study, with smoking cessation, commonly advised for pneumothorax cases following the first episode in clinical practice. Consequently, smoking cessation following the FE did not demonstrate efficacy in extending the RFS when the presence of bulla–bleb structures on tomography and the DCT were adjusted. The presence of bulla–bleb structure on CT was not found to have an impact on the RFS when the effect of the DCT and smoking status after the FE were adjusted. It was found that the risk of recurrence decreased by 19.7% with a one day increase in DCT (HR = 0.803; *p* = 0.002) (Table 5). The results of the analyses were presented as flowchart (Figure 1).

**TABLE 5:** Multiple regression model established with statistically significant values.

				95.0% CI for exp(B)	
Characteristics	B	Sig.	Exp(B)	Lower	Upper
Smoking after first episode	0.411	0.138	1.509	0.876	2.599
Bullae‐bleb on CT	0.432	0.114	1.541	0.901	2.635
Duration of chest tube drainage (day)	−0.219	***0.002***	0.803	0.698	0.924

*Note:* Statistically significant results are indicated in bold and italics.

## 4 Discussion

This study examined the factors influencing recurrence in PSP cases from various perspectives. The hypothesis that the process may be associated with inflammation has been previously proposed in the literature. There are studies correlating inflammation parameters with the number of recurrences [3, 5]. These two studies offer definitive examples indicating a possible correlation between spontaneous pneumothorax and inflammation. Our study did not examine the correlation between inflammation parameters and the heightened frequency of recurrences. It aimed to determine whether inflammatory parameters could serve as predictors of recurrence time. We investigated the potential for recurrence by analyzing basic blood parameters of patients experiencing a FE and assessed whether surgical intervention can be advised during the FE based on the findings. Our findings indicate that there is no significant correlation between inflammatory parameters and the risk of recurrence or RFS. In our study, which aimed to practically predict recurrence based on inflammatory parameters, we were unable to obtain statistically significant results for this variable. Although the literature supports a relationship between inflammation and recurrence, our findings suggest that this association does not necessarily translate into a predictive value through standard inflammatory markers. Several factors may have contributed to the negative findings, including the timing of blood sample collection and the potential inadequacy of the selected inflammatory parameters. For instance, blood samples taken at the time of discharge, rather than at initial admission, might have yielded more meaningful results. Alternatively, pleural lavage followed by cellular analysis could have provided further insight. Although such approaches were not feasible because of the nature of our study, we believe that the relationship between inflammation and pneumothorax recurrence remains a subject worthy of further investigation.

Numerous factors have been examined in literature to predict recurrence. A study examining the correlation between pneumothorax volume and recurrence risk categorized cases based on their pneumothorax volumes as either below or above 50%. The pneumothorax volume in smokers exceeded 50%, and cases of prolonged air drainage and recurrence were observed more frequently in these cases. This study employed two different methods for calculating pneumothorax volume—the Kircher and Swatzel methods [9]. Our study employed the Collins method and BTS classifications, revealing no significant correlation between the initial pneumothorax volume and the RFS. Another study examined the correlation between pneumothorax volume and recurrence, evaluating 128 cases of PSP. A significant correlation was identified between large pneumothorax volume and prolonged air drainage. This study also assessed the impact of bullous structures on the risk of recurrence. Patients without bullae and those with either single or multiple bullae were compared, and the quantity of bullae was not assessed as a prognostic indicator regarding recurrence [10]. This study yields comparable results to our study regarding the quantity of bullae, but it was conducted to assess the risk of recurrence. In our study, on the other hand, factors influencing early recurrence were investigated through a recurrence‐free survival analysis. Furthermore, our study assessed the presence or absence of visible bullae on CT in cases of pneumothorax which constitutes one of our clinical challenges. Consequently, a markedly earlier recurrence was noted in patients exhibiting visible bullae on CT. More recent studies in the literature have also focused on recurrence‐free survival analysis, supporting our findings. In a study conducted in 2015, the dystrophy severity score was used, and it was concluded that patients with at least one bulla who did not undergo surgical treatment, or those with high dystrophy scores, had a higher risk of early recurrence. Therefore, surgical treatment was recommended at the first episode, and conservative management was discouraged in such patients [11]. Similarly, another study from 2022 that also used the dystrophy score followed one‐year recurrences in non‐surgically treated cases and reported that high dystrophy scores were associated with early recurrence [12].

Our study incorporates both the recency of modern recurrence‐free survival analyses and the practicality of the older studies' simplistic approach such as the mere presence or absence of bullae, and demonstrates that the presence of bullae alone is associated with early recurrence. When informing patients who are recommended surgical treatment for the first episode for any indication, knowing that bullae on tomography may be associated with early recurrence may positively change the patient's decision.

A meta‐analysis examined the impact of smoking on recurrence in patients undergoing VATS. The meta‐analysis results indicated that smoking was not considered a significant prognostic factor for recurrence. This study exclusively assessed the pre‐episode smoking status of the patients [13]. Our study evaluated the patients' smoking histories both before the pneumothorax and following the FE. The amount of consumption prior to the episode was not identified as a significant factor influencing recurrence; however, smoking after the FE was considered risky regarding early recurrence in univariate analysis. A study investigating the factors influencing pneumothorax recurrence concluded that smoking was not considered a significant contributor to recurrence. The research revealed young age and the administration of tube thoracostomy at the FE as important risk factors for recurrence. Similar to our study, this research involved patients who did not receive surgical intervention. No patients underwent VATS wedge resection in the cases examined, and the authors found that patients who underwent tube thoracostomy experienced recurrence more frequently within three years compared to those who did not [14]. Although studies investigating the relationship between smoking and recurrence exist in the literature, they remain limited in number. These studies have also emphasized that inconsistent results were obtained because of the lack of a clear definition of the smoking variable and the scarcity of such research [15]. According to our research, our study is one of the few that clearly defines and investigates smoking status after the episode. In a study conducted in 2022, the authors investigated the factors affecting recurrence and emphasized that among patients who were managed conservatively during their first episode, those who did not quit smoking afterward experienced significantly more frequent recurrences [16]. The design and results of this study are supportive of our findings. Although the primary aim of that study was to evaluate the relationship between conservative versus surgical management and recurrence, the outcomes of patients who underwent conservative treatment were similar to ours. In line with this, the presence of bullae or blebs on CT imaging, in addition to continuing to smoke after the episode, was another finding consistent with our study.

In our study, treatment approaches were also evaluated. However, since the patient population consisted of those who did not undergo surgical treatment, whether the patients were managed with tube thoracostomy or oxygen therapy was not significantly associated with increased recurrence. In addition to this expected result, we found that prolonged tube thoracostomy (DCT) reduced the risk of recurrence in non‐surgically treated patients.

A recent study (2024) compared postoperative DCT in surgically treated PSP cases. The risk of recurrence was elucidated increasing with a prolonged air leak following surgical treatment. This study assesses the risk of recurrence following surgical intervention. Consequently, the authors deemed it a natural outcome that the patient experiencing prolonged postoperative air leakage was identified as being at risk for recurrence [17]. Our study examined the factors influencing the recurrence in patients who did not receive surgical intervention. Our findings indicate that DCT significantly extended recurrence‐free survival and reduced the recurrence risk by 19.7%. Although the authors of the referenced study anticipated early recurrence in patients with extended air leakage, our research contends that prolonged DCT reduces the risk of recurrence in patients undergoing tube thoracostomy as the initial intervention in the absence of surgical treatment. A 2020 study reported that, among first‐episode spontaneous pneumothorax patients managed with tube thoracostomy, prolonged hospitalization, and extended duration of DCT were significantly associated with a reduced risk of recurrence. These findings support our study both in terms of results and by highlighting a current clinical interest [18]. According to our research, it is the only study investigating the effect of chest tube duration on recurrence time in a similar population. The duration of drainage ranges from 2 to 15 days in our study, with patients experiencing a prolonged drainage period exceeding 7 days being those who initially declined surgical intervention during the FE. In our practice, chest tubes are removed on the second day after the end of air leak. This protocol, together with the prolonged duration of tube thoracostomy, may result in improved expansion, even at the millimeter level, increased adhesions on all surfaces, and a reduced risk of recurrence. Our population comprises patients who refuse surgery despite prolonged air drainage and are observed with a chest tube, making it a significant cohort for assessing the challenges these patients may encounter in the future.

The literature contains studies assessing RFS in pneumothorax, similar to our investigation. Nonetheless, these studies primarily examine the postoperative effectiveness of surgical techniques, instruments, and air leakage barriers [19, 20, 21, 22]. This study aimed to analyze the timing and risk of recurrence in first‐episode PSP patients who did not get surgical treatment, and to identify the risk factors associated with early recurrences in this cohort.

The main limitation of our study is its retrospective design. Despite being a study conducted across two centers; our case number was restricted to 117 because of the exclusion of severe cases. Nonetheless, we believe that this selected population will adequately represent patients who have not received surgical treatment. A further limitation of the study was that post‐episode smoking was assessed solely in categorical terms. Evaluating the amount of tobacco consumption may provide more precise data for assessing recurrence risk and RFS.

In conclusion, in patients experiencing their first episode of PSP, smoking post‐episode, and the identification of a visible bullae–bleb structure on CT are risk factors for an earlier recurrence.

In patients with first episode PSP, the presence of bullae–bleb on CT may indicate a potential for early recurrence, supporting a recommendation for surgery.

Patients experiencing the first episode of PSP who decline surgical intervention or lack indications for surgery may be advised that continued smoking post‐discharge could lead to early recurrence.

Prolonged duration of chest tube drainage reduces the risk of earlier recurrence in first‐episode PSP cases that have not undergone surgical intervention. This finding suggests that extended drainage is not a disadvantageous outcome for cases hesitant to undergo surgical treatment.

## Author Contributions

**Sercan Aydin:** conceptualization, writing – original draft. **Seda Kahraman Aydin:** writing – review and editing. **Dilara Gursoy:** data curation, writing – original draft. **Ayse Gul Ergonul:** methodology, resources. **Tevfik Ilker Akcam:** resources. **Kutsal Turhan:** resources. **Ufuk Cagirici:** supervision. **Alpaslan Cakan:** supervision. **Omer Faruk Dadas:** formal analysis.

## Ethics Statement

Izmir Democracy University Buca Seyfi Demirsoy Education and Research Hospital Clinical Research Ethics Committee were approved for this study (approval number 2024/242). All methods were performed in accordance with the relevant guidelines and regulations.

## Consent

Informed consent was obtained from all individual participants included in the study.

## Conflicts of Interest

The authors declare no conflicts of interest.
